# Dermatology Match Trends From 2022–2025 Using the Texas Seeking Transparency in Application to Residency Database

**DOI:** 10.7759/cureus.107744

**Published:** 2026-04-26

**Authors:** Aya Salem, Adam Ali, Layla Ali, Hannah Wolfson, Ahmed Salem, Sydney Hou, Angela Mihalic, Michael S Wong

**Affiliations:** 1 Medicine, California Northstate University College of Medicine, Elk Grove, USA; 2 Otolaryngology, California Northstate University College of Medicine, Elk Grove, USA; 3 Pediatrics, University of Texas Southwestern Medical Center, Dallas, USA; 4 Surgery/Plastic Surgery, California Northstate University College of Medicine, Elk Grove, USA

**Keywords:** dermatology, match trends, nrmp, step 2, texas star database

## Abstract

Background: Dermatology is one of the most competitive residency specialties in the United States. In 2022, the United States Medical Licensing Examination (USMLE) Step 1 transitioned from numerical scoring to pass/fail. Additional changes, including preference signaling and interview caps, were introduced during this period. This study analyzes dermatology residency application trends from 2022-2025 using the Texas Seeking Transparency in Application to Residency (STAR) database.

Methods: A retrospective database analysis was conducted using the Texas STAR dataset, a national, multi-institutional repository of self-reported residency application metrics. Dermatology applicants from participating U.S. medical schools between 2018 and 2025 were included, with particular focus on the 2022-2025 application cycles surrounding the transition of the USMLE Step 1 to pass/fail scoring.

Applicant characteristics were analyzed across multiple domains, including the number of programs applied to, USMLE Step 2 Clinical Knowledge (CK) scores, research experiences, peer-reviewed publications, leadership activities, clerkship honors, and participation in a dedicated research year. Descriptive statistics were used to summarize these variables.

Comparisons across application cycles were performed to assess changes over time. Continuous variables were analyzed using Wilcoxon rank-sum tests, while categorical variables were compared using chi-square tests. Statistical significance was defined as a p-value less than 0.05.

Results: Applications per applicant declined substantially after 2023, with mean applications decreasing from approximately 82 in 2022-2023 to 45 in 2024 and 29 in 2025 (p < 0.01). Step 2 CK scores remained stable across all cycles with no significant differences (p = 0.62). Research productivity showed a modest decline following 2023. Mean peer-reviewed publications decreased from 7.77 in 2023 to 5.55 in 2024 (p < 0.01), and leadership experiences declined from 5.57 to 3.69 during the same period (p < 0.01). Research year participation remained stable across cycles (p = 0.76). Clerkship honors showed no significant differences between years (p = 0.52).

Conclusion: Recent dermatology applicants appear to be submitting fewer applications and reporting fewer research and leadership experiences while maintaining stable academic performance. These trends likely reflect structural changes in the residency application process, including preference signaling and efforts to reduce application inflation following the Step 1 pass/fail transition. Despite these shifts, core academic metrics such as Step 2 CK scores and clerkship honors remain consistent, suggesting continued emphasis on academic performance in dermatology residency selection.

## Introduction

Dermatology has long been recognized as one of the most competitive residency specialties in the United States. Every year, the number of qualified medical students far exceeds the number of available positions, making the match rate among the lowest across all disciplines. Applicants participate through the Electronic Residency Application Service (ERAS) and are matched using the National Resident Matching Program (NRMP). In recent years, many programs have also adopted preference signaling, allowing students to indicate a limited number of programs they are most interested in. Because of the field’s competitiveness, programs tend to look at a mix of quantitative, such as test performance and clinical evaluations, and qualitative metrics, such as leadership and community service.

Previous studies have shown that dermatology match success often correlates with a combination of strong standardized exam performance and substantial research productivity [1]. Earlier analyses from Charting Outcomes in the Match and several studies using the Texas Seeking Transparency in Application to Residency (STAR) database have demonstrated that applicants who match into dermatology usually report higher Step 1 and Step 2 Clinical Knowledge (CK) scores, more publications, and greater research involvement than unmatched peers [2]. These findings have reinforced the idea that dermatology favors academically oriented students with a clear record of dedication to the field [3]. Leadership and volunteer experiences, as well as honors in clinical clerkships, have also been cited as important factors that can distinguish otherwise similar applicants [4]. However, as the application process has evolved, it is not entirely clear whether these indicators hold the same weight in medical education and residency selection [2].

A major shift in residency selection occurred in early 2022 when the United States Medical Licensing Examination (USMLE) Step 1 transitioned from a numerical score to a pass/fail reporting system [5]. The class of 2024 was the first group of medical students to apply for residency without a Step 1 score, and both applicants and program directors were forced to rethink how they assess qualifications. In the absence of a standardized numerical cutoff, many programs began relying more heavily on Step 2 CK performance, which remains numerically scored, as well as on other indicators of academic ability such as research productivity and clerkship evaluations [5]. For competitive specialties like dermatology, this change raised questions about how programs would continue to differentiate among a large pool of high-achieving applicants. Early commentaries suggested that Step 2 CK might become the new screening benchmark, while qualitative factors, such as letters of recommendation and personal statements, could take on a greater role [6]. At the same time, initiatives such as preference signaling and holistic review were introduced in an attempt to overcome application inflation and promote a fairer, more efficient selection process [6]. In 2024, program signaling in dermatology was limited to three gold and 25 silver tokens [6].

Although these changes have been widely discussed, few studies have examined how dermatology application trends have actually shifted in the years since the Step 1 score change. The Texas STAR database provides a particularly valuable resource for this purpose. It compiles multi-institutional, self-reported application data from medical students nationwide, including exam scores, research activities, and interview outcomes. Previous Texas STAR analyses have offered insight into broad applicant behaviors across multiple specialties, yet dermatology-specific data from 2022 through 2025 have not been formally analyzed. Because this time frame spans both the final years of numerical Step 1 scoring and the early years of the pass/fail system, it offers a rare opportunity to observe how applicant characteristics and strategies have evolved in real time.

The goal of the present study is to examine dermatology residency application trends from 2022 through 2025 using the Texas STAR dataset. By comparing various application metrics before and after the Step 1 policy change, this analysis aims to capture how the specialty’s applicant profile has shifted in the aftermath. Understanding these changes is valuable not only for medical students preparing their residency applications but also for program directors seeking to design fair and effective selection processes. Ultimately, examining these patterns may help clarify whether the intended goals of recent reforms, to promote holistic review and encourage meaningful engagement in extracurriculars, are being realized within one of the most competitive specialties in graduate medical education.

## Materials and methods

Study design and data Source

This retrospective database study used data from the Texas STAR program, a national, multi-institutional repository of self-reported residency application data [2]. The Texas STAR program collects applicant-reported information from participating United States medical schools regarding application characteristics, academic metrics, and match-related outcomes. Prior studies have used the Texas STAR database to evaluate dermatology residency application trends and outcomes [2]. The Texas STAR dataset is limited to participating institutions and is not reflective of national standards. Data reflects anywhere from 23-38% of national medical schools. As this study involved human participants through the use of de-identified, retrospective data from a national database, institutional review board (IRB) exemption was granted under protocol number 2408-02-169. 

Study population

Dermatology applicants from participating United States medical schools were identified within the Texas STAR database from the 2018 through 2025 application cycles. Although broader descriptive trends from 2018 to 2025 were reviewed, the primary analysis focused on the 2022-2025 cycles to evaluate applicant characteristics surrounding the transition of USMLE Step 1 from numerical scoring to pass/fail reporting [5,6]. Applicants with incomplete data for the variables of interest were excluded from individual analyses as applicable. Reapplicants were included in the dataset without the ability to distinguish between them. DO and MD applicants were labeled accordingly, and both were included in the dataset.

Variables

Variables analyzed included number of programs applied to, USMLE Step 2 CK score, research year participation, number of research experiences, number of peer-reviewed publications, number of leadership experiences, and number of clerkship honors. Variables were self-reported after matching. All applicants who matched were pooled together.

Step 2 CK scores were analyzed according to the scoring format available within the Texas STAR dataset. These score ranges were determined by National Board of Medical Examiners (NBME) percentile groupings (i.e., 250-4, etc.). Research year participation was treated as a categorical variable, while the remaining variables were analyzed as continuous or ordinal count-based measures.

Statistical analysis

Descriptive statistics were calculated for each application year and reported as mean, median, and SD for continuous variables, and as frequencies for categorical variables. Comparisons between 2023 and 2024 were performed to assess immediate changes during the post-Step 1 transition period. Additional pooled comparisons were performed between 2022/2023 and 2024/2025 to evaluate broader differences before and after this transition.

Because the data were non-normally distributed and derived from independent samples, Wilcoxon rank-sum tests were used to compare continuous variables across years. Chi-square testing was used to compare categorical variables, including research year participation. Statistical significance was defined as a two-sided p value < 0.05. Analyses were conducted in R version 4.3.3.

## Results

Overview

From 2018 to 2025, dermatology applicants in the Texas STAR database demonstrated relatively stable academic metrics but notable changes in application behavior and extracurricular profile over time. The most pronounced recent trend was a marked decline in the number of applications submitted per applicant after 2023. In contrast, Step 2 CK scores and honors remained relatively stable across cycles. Research productivity and leadership involvement showed modest declines in the more recent years.

Descriptive statistics by year

Applicant characteristics by application year are summarized in Table 1.

**Table 1:** Dermatology applicant characteristics by application year CK: Clinical Knowledge

Year	Applications, Mean ± SD	Step 2 CK, Mean ± SD	Research Year, n (%)	Research Experiences, Mean ± SD	Peer-Reviewed Publications, Mean ± SD	Leadership Experiences, Mean ± SD	Honors Rotations, Mean ± SD
2025	28.66 ± 17.81	12.13 ± 2.47	35/96 (36.5%)	7.97 ± 4.38	6.75 ± 4.12	4.49 ± 2.47	4.31 ± 2.28
2024	45.40 ± 33.38	12.13 ± 2.20	35/129 (27.1%)	5.99 ± 3.96	5.55 ± 4.14	3.69 ± 2.19	4.44 ± 2.44
2023	83.85 ± 46.62	12.33 ± 2.15	47/136 (34.6%)	6.86 ± 3.45	7.77 ± 3.92	5.57 ± 2.93	4.26 ± 2.59
2022	81.95 ± 47.19	12.20 ± 2.10	51/163 (31.3%)	6.80 ± 3.39	7.35 ± 4.10	5.81 ± 3.05	4.10 ± 2.70
2021	75.90 ± 43.58	12.52 ± 2.06	33/110 (30.0%)	6.45 ± 3.18	7.82 ± 4.03	5.63 ± 3.22	4.72 ± 2.40
2020	86.05 ± 41.78	12.08 ± 2.09	42/131 (32.1%)	5.52 ± 3.21	6.49 ± 4.39	5.26 ± 3.43	4.80 ± 2.35
2019	77.21 ± 44.23	12.33 ± 2.18	31/111 (27.9%)	6.19 ± 3.25	5.89 ± 3.90	4.84 ± 3.29	5.00 ± 2.40
2018	85.98 ± 33.84	12.20 ± 2.22	33/93 (35.5%)	5.92 ± 3.25	5.72 ± 3.89	5.10 ± 3.08	5.31 ± 2.25

Application Volume

The mean number of applications submitted per applicant was high in 2022 and 2023, at 81.95 and 83.85, respectively, but declined sharply in 2024 and 2025 to 45.40 and 28.66. This reduction was statistically significant for both the 2023 versus 2024 comparison (p = 5.513 × 10⁻¹¹) and the pooled 2022/2023 versus 2024/2025 comparison (p < 2.2 × 10⁻¹⁶) (Table 2).

**Table 2:** Statistical comparisons of key dermatology applicant metrics CK: Clinical Knowledge

Comparison	Variable	Statistical Test	Test Statistic	p-value	
2023 vs 2024	Applications	Wilcoxon rank-sum test	W = 12860	5.513 × 10⁻¹¹	
2023 vs 2024	Step 2 CK	Wilcoxon rank-sum test	W = 9156.5	0.3379	
2023 vs 2024	Research Year	Chi-square test	χ² = 1.3791	0.2403	
2023 vs 2024	Research Experiences	Wilcoxon rank-sum test	W = 10222	0.006354	
2023 vs 2024	Publications	Wilcoxon rank-sum test	W = 11208	1.237 × 10⁻⁵	
2023 vs 2024	Leadership Experiences	Wilcoxon rank-sum test	W = 11230	3.55 × 10⁻⁹	
2023 vs 2024	Honors Rotations	Wilcoxon rank-sum test	W = 7128.5	0.6924	
2022/2023 vs 2024/2025	Applications	Wilcoxon rank-sum test	W = 51346	< 2.2 × 10⁻¹⁶	
2022/2023 vs 2024/2025	Step 2 CK	Wilcoxon rank-sum test	W = 33693	0.6157	
2022/2023 vs 2024/2025	Research Year	Chi-square test	χ² = 0.095872	0.7568	
2022/2023 vs 2024/2025	Research Experiences	Wilcoxon rank-sum test	W = 34770	0.2999	
2022/2023 vs 2024/2025	Publications	Wilcoxon rank-sum test	W = 39676	4.919 × 10⁻⁵	
2022/2023 vs 2024/2025	Leadership Experiences	Wilcoxon rank-sum test	W = 43071	2.524 × 10⁻¹²	
2022/2023 vs 2024/2025	Honors Rotations	Wilcoxon rank-sum test	W = 27861	0.5164	

Step 2 CK Performance

Step 2 CK scores remained stable across the study period, with mean values clustered around 12 on the Texas STAR scoring scale. There were no significant differences between 2023 and 2024 (p = 0.3379) or between pooled 2022/2023 and 2024/2025 groups (p = 0.6157) (Table 2).

Research Year Participation

Research year participation remained relatively consistent across recent cycles. In 2025, 36.5% of applicants reported a research year, compared with 27.1% in 2024, 34.6% in 2023, and 31.3% in 2022. These differences were not statistically significant in either the 2023 versus 2024 comparison (p = 0.2403) or the pooled 2022/2023 versus 2024/2025 comparison (p = 0.7568) (Table 2).

Research Experiences and Publications

Research experiences showed a modest decline after 2023. Mean research experiences fell from 6.86 in 2023 to 5.99 in 2024, representing a significant difference (p = 0.006354), although pooled comparisons were not significant (p = 0.2999). Peer-reviewed publication counts also declined, decreasing from 7.77 in 2023 to 5.55 in 2024 (p = 1.237 × 10⁻⁵), with this trend remaining significant in pooled comparisons (p = 4.919 × 10⁻⁵) (Table 2).

Leadership and Clerkship Honors

Leadership experiences declined substantially after 2023. The mean number of leadership experiences decreased from 5.57 in 2023 to 3.69 in 2024 (p = 3.55 × 10^-9^), and the pooled comparison between 2022/2023 and 2024/2025 was similarly significant (p = 2.524 × 10^-12^).

In contrast, clerkship honors remained stable across years. Mean honors ranged from approximately 4 to 5 across all recent cycles, with no significant difference between 2023 and 2024 (p = 0.6924) or between 2022/2023 and 2024/2025 (p = 0.5164).

## Discussion

One of the clearest findings in this analysis was the marked decline in the number of applications submitted per applicant after the 2023 cycle. This trend is consistent with national data showing that, in specialties adopting program signals, the average number of applications per applicant and per program fell between the 2023 and 2024 ERAS cycles, particularly in large-signal and two-tier signaling specialties [7]. Rather than reflecting a sudden decrease in applicant competitiveness, this pattern likely reflects structural changes in the residency application ecosystem, including the expansion of preference signaling and coordinated efforts by the Association of American Medical Colleges (AAMC), specialty organizations, and advisors to curb application inflation and encourage more targeted application strategies [8]. Studies in otolaryngology and other signal-heavy fields have similarly demonstrated that implementation of preference signals is associated with fewer applications received per program and that applicants increasingly recognize diminishing returns from applying far beyond the number of signals they can send [9]. Together, these changes suggest that applicants are becoming more selective about where they apply, using signals, advisor guidance, and perceived interview yield to focus their lists rather than indiscriminately maximizing application volume [7-9].

The decline in application volume in the later cycles may also suggest that applicants perceived the process as becoming more targeted and strategic. Applicants in the preference-signaling era are encouraged to use a limited number of signals, geographic preference designations, and tailored application strategies to convey genuine interest to selected programs rather than applying indiscriminately [10,11]. Early evaluations of program signaling describe it as a tool to mitigate application inflation by nudging applicants to think more critically about where they apply and to concentrate efforts on programs where they perceive the best fit [12].

Despite concerns that the transition of USMLE Step 1 to pass/fail reporting would lead applicants to compensate by dramatically increasing other measurable achievements, survey data and performance reports suggest only modest shifts rather than abrupt inflation in Step 2 CK scores [13,14]. In dermatology, both residents and program directors anticipated greater emphasis on Step 2 CK, research, and extracurricular activities after the Step 1 change, but available national Step 2 performance data do not show a sudden, large-scale score escalation during the early post-Step 1 era [13,14]. This pattern is consistent with our finding that Step 2 CK scores in this dermatology applicant cohort remained relatively stable across study years, implying that academic performance has remained consistently high even as the structure of the application process has changed.

The stability in Step 2 CK scores within this sample may therefore indicate that Step 2 did not undergo a dramatic inflationary shift during the early post-Step 1 period, at least among dermatology applicants. Instead, applicants appear to have maintained a similar academic profile while adapting other aspects of their application strategy, such as where they apply, how they deploy preference signals, and how they frame geographic and program-specific interests, in response to evolving selection practices [14].

Research productivity showed a modest decline in the post-2023 period, particularly with regard to peer-reviewed publications. This may reflect reduced pressure to build increasingly inflated research portfolios, or it may indicate that applicants are reallocating time toward other components of the application that are less easily captured in this dataset. Importantly, research year participation did not significantly change across cycles. This suggests that while total publications and research experiences may have declined modestly, the decision to take a dedicated research year remains a relatively stable strategy among dermatology applicants.

The decline in leadership experiences is also notable. One interpretation is that applicants may no longer feel compelled to maximize every extracurricular category in the same way as before. Another possibility is that recent applicants are reporting fewer but more meaningful leadership roles rather than accumulating a greater number of nominal positions.

Clerkship honors also remained stable, which may indicate that clinical performance continues to serve as a consistent marker of competitiveness in dermatology. Even amid broader reforms, strong academic and clinical achievement appears to remain central to the applicant profile.

Taken together, these findings suggest that recent reforms may be accomplishing part of their intended purpose. Applicants appear to be submitting fewer applications and reporting fewer activities in some categories without evidence of declining academic performance. This could reflect a gradual movement toward a less inflated and potentially more efficient application process.

At the same time, these findings should be interpreted cautiously. Because this study evaluates applicant-reported metrics rather than program selection behavior directly, it cannot determine whether programs themselves changed the weight assigned to different application components. Additional work examining interview offers, match outcomes, and program director preferences would help clarify whether these observed trends correspond to meaningful changes in residency selection.

Limitations

This study has several limitations. First, the Texas STAR database is based on self-reported applicant data and is, therefore, subject to recall bias, reporting bias, and variability in how applicants interpret survey items. Second, participation in Texas STAR is voluntary, which introduces the possibility of selection bias and limits generalizability to all United States dermatology applicants. Response rates also vary by institution and year, and the dataset may not fully capture the national applicant pool. Third, Step 2 CK was analyzed according to the scoring format available within the dataset rather than as raw numerical scores, which may reduce interpretability. Finally, this analysis is descriptive and cannot establish causality; observed changes may be associated with multiple concurrent reforms, including Step 1 pass/fail, preference signaling, and shifting advising practices.

Human subjects: This study involved human participants through the use of de-identified, retrospective data from a national database. Institutional Review Board (IRB) exemption was granted under protocol number 2408-02-169, as the study used existing, de-identified data and did not involve direct interaction with participants or identifiable private information.

## Conclusions

Dermatology applicants in the Texas STAR database demonstrated substantial changes in application behavior between 2022 and 2025. The most notable trend was a sharp decline in the number of programs applied to after 2023, accompanied by modest decreases in peer-reviewed publications and leadership experiences. In contrast, Step 2 CK scores, research year participation, and clerkship honors remained largely stable.

These findings suggest that recent applicants may be pursuing a more selective and less inflated application strategy while maintaining a similarly strong academic profile. This is important because it may reflect the early effects of residency application reforms aimed at improving efficiency and encouraging more meaningful evaluation of applicants. Further studies are needed to determine whether these trends persist and whether they translate into changes in interview distribution, match outcomes, and residency selection practices in dermatology.
